# Alpha-1-acid glycoprotein as potential biomarker for alpha-fetoprotein-low hepatocellular carcinoma

**DOI:** 10.1186/1756-0500-3-319

**Published:** 2010-11-23

**Authors:** Indra Bachtiar, Valentine Kheng, Gunawan A Wibowo, Rino A Gani, Irsan Hasan, Andri Sanityoso, Unggul Budhihusodo, Syafruddin AR Lelosutan, Ruswhandi Martamala, Wenny A Achwan, Soewignyo Soemoharjo, Ali Sulaiman, Laurentius A Lesmana, Susan Tai

**Affiliations:** 1 Proteomic Division, Mochtar Riady Institute for Nanotechnology, Lippo Karawaci, Tangerang, 15811, Indonesia; 2 Hepatology Division, Department of Internal Medicine, Faculty of Medicine University of Indonesia, Jakarta, Indonesia; 3 Gastroentero-Hepatology Division, Department of Internal Medicine, Gatot Soebroto Hospital, Jakarta, Indonesia; 4 Department of Internal Medicine, Mataram General Hospital, Mataram, Indonesia; 5 Department of Hepatology, Klinik Hati "Prof. Ali Sulaiman", Jakarta, Indonesia

## Abstract

**Background:**

The outcome of patients with hepatocellular carcinoma (HCC) remains poor because of late diagnosis. We determined the performances of α -1-acid glycoprotein (AAG) and des-γ-carboxy prothrombin (DCP) for the diagnosis of HCC, especially for α-fetoprotein (AFP)-low HCC.

**Methods:**

Of the 220 patients included in this retrospective study, 124 had HCC, and 61 (49%) of these were AFP-low HCC (AFP ≤ 20 ng/mL). The remaining 96 patients, including 49 with chronic hepatitis B or C and 47 with cirrhosis, were considered as control. Plasma AAG was analyzed using high performance liquid chromatography (HPLC) and confirmed using Western blot technique.

**Results:**

When all patients with HCC were evaluated, the area under receiver operating characteristic (ROC) curves for AAG (0.94, 95% CI: 0.91-0.97) and DCP (0.92, 95% CI: 0.88-0.95) were similar (*P *= 0.40). AAG had better area under ROC curve (0.96, 95% CI: 0.94-0.99) than DCP (0.87, 95% CI: 0.81-0.93) for AFP-low HCC (*P *< 0.05). At the specificity 95%, the sensitivity of AAG was higher in AFP-low HCC than in AFP-high HCC (82% and 62%, respectively). In contrast, higher sensitivity was obtained from DCP in discriminating HCC patients with low AFP than that in high AFP (57% and 90%, respectively).

**Conclusion:**

Our cross-sectional study showed that AAG was better performance in diagnosing HCC patients with low AFP, while DCP did better in those with high AFP.

## Background

Hepatocellular carcinoma (HCC) is one of the most common malignancies in the world [1]. Until recently, AFP has been the most widely used plasma marker for diagnosis, surveillance, and as a prognostic indicator of HCC patients' survival [2]. Several studies indicated that high plasma levels of AFP are related to poor prognosis, as well as histologic grade of malignancy [3]. Those with high plasma AFP level at the time of HCC diagnosis have more unfavourable outcomes compared to patients with low AFP level [4]. However, it has been recognized that AFP has a low sensitivity in detection of HCC, and that AFP level often increases in the absence of HCC [5].

To date, several tumor markers have been proposed as complement or substitute for AFP in HCC diagnosis. Recently, *Lens culinaris *agglutinin-reactive fraction of AFP (AFP-L3) [6] and DCP [7] have been approved by the Food and Drug Administration as plasma markers for HCC. AFP-L3 can be detected in the plasma of patients with small tumors [8,9]. However, for the diagnosis of early stage of HCC, AFP-L3 [10] and DCP [10,11] are less sensitive than AFP for the diagnosis of early and very early stage HCC. DCP has not been used in Indonesia and it could be a potential biomarker for diagnosis of HCC. Therefore a cross-sectional study is required to determine the role of DCP in the diagnosis of HCC in the Indonesian population. Another potential biomarker for HCC is AAG. AAG is an acute phase protein, synthesized predominantly in the liver. Cytokines can cause plasma AAG level to increase as part of an inflammatory response [12]. The plasma level of AAG has been suggested to be a potential marker for diagnosing cirrhosis and HCC [13]. Recently we have shown that combination of AAG and AFP improves the accuracy of HCC diagnosis [14].

Given the rising incidence of HCC in Indonesia and lack of data on the clinical utility of these two tumor markers (AAG and DCP) for detecting the presence of HCC, we selected 220 chronic liver patients, with and without HCC. This study is a further step in order to assess the feasibility of AAG to diagnose AFP-low HCC. This study has limitations, and not designed to assess the treatment and follow-up of the screen-detected patients. Our specific aims were to: (i) define the level of each tumor marker with the best sensitivity, specificity, accuracy, positive predictive value (PPV) and negative predictive value (NPV) for diagnosis of HCC; and (ii) to evaluate the clinical utilities of AAG and DCP in HCC patients with low AFP (≤ 20 ng/mL) and high AFP (> 20 ng/mL), especially in attempt to find the best biomarker for diagnosing HCC patients with low AFP.

## Methods

### Patients

A total of 220 plasma samples were collected between January 2006 and January 2009 from patients in four participating hospitals, Cipto Mangunkusumo Hospital, Gatot Subroto Army Hospital, Ali Sulaiman Liver Clinic Center (Jakarta, West Indonesia) and Hepatitis Laboratory Mataram (West Nusa Tenggara, East Indonesia). Demographic and clinical information of each patient including age, gender, and cause of disease were obtained from chart review. All patients gave informed consents to participate in the study, and the Institutional Review Board of each participating institution approved the protocol.

The patients were divided into three groups: (i) control (non-malignant disease), either chronic hepatitis patients with HBV/HCV infection (*n *= 49) or cirrhotic patients (*n *= 47); (ii) AFP-low HCC (*n *= 61); and (iii) AFP-high HCC (*n *= 63). Etiology of underlying liver disease was attributed to HBV based on detection of HBsAg in plasma via a commercial assay and HCV based on detection of hepatitis C antibody/hepatitis C virus RNA in plasma. The presence of cirrhosis was defined by histological measurement or non-histological by evidence of portal hypertension in the presence of chronic liver disease. Diagnosis of HCC relied on the presence of a malignant liver nodule, as established by imaging techniques. Fine needle aspiration biopsy procedure reconfirmed the diagnosis of HCC for sample in which the AFP concentration was low. Subjects with HIV co-infection or autoimmune hepatitis were excluded in this study. None of the subjects was taking vitamin K at time of the study.

### Biomarkers measurements

Blood samples were collected in pyrogen-free tube (BD Vacutainer, Plymouth, UK) with ethylene diamine tetra-acetic acid as an anticoagulant. A 10 mL blood sample was drawn from each subject, and plasma was stored at -80°C until AFP, AAG, and DCP testing.

The quantitative measurement of plasma AFP or DCP was performed using a commercially available enzyme-linked immunosorbent assay (ELISA) kit from Amgenix International Inc. (San Jose, CA, USA) or from Diagnostica Stago (Asnières-sur-Seine, France), respectively, according to the manufacturers' instructions. The minimum detectable concentration for both AFP and DCP were 2.0 ng/mL.

Measurement of plasma AAG concentration was performed according to our previous report [14]. Briefly, the plasma AAG level was detected via the Agilent 1200 HPLC system. A 200 μL of crude plasma was diluted with 600 μL of Buffer A (Agilent, Santa Clara, CA). An amount of 20 μL of the diluted plasma was depleted on a Multiple Affinity Removal Column (Agilent; 4.6 mm × 50 mm) according to the manufacturer's instructions. The total volume of depleted plasma collected at retention time between 2 and 6 min was approximately 1 mL. The depleted plasma was fractionated by column mRP-C18 (Agilent; 4.6 mm × 50 mm), operated at flow rate 0.75 mL/min and mobile phase comprised of two solvents: A: water/0.1% trifluoroacetic acid (TFA) and B: Acetonitrile/0.08% TFA. The AAG fraction was detected between retention times at 14.5-16 minute. Quantification of plasma AAG concentration was performed using ovalbumine as an external standard and for calibration sample. Measurement of the standard was calculated based on peak area integration according to the Agilent Chemstation protocol.

### Western blot

The result from HPLC analysis was validated using Western blot. We selected 8 samples randomly from each group, chronic hepatitis, cirrhosis, AFP-low HCC, and AFP-high HCC, respectively. An amount of 25 μL plasma was depleted using Multiple Affinity Removal Column (Agilent). The low-abundant protein fractions were collected and concentrated to 100 μL. Then, 10 μL of the concentrated fractions were mixed with Laemmli sample buffer and applied to 12% SDS-PAGE gels. Proteins in the gels were transferred to 0.45 μm nitrocellulose membranes. The membranes were blocked using 5% skim milk in TBS-T (20 mM Tris-HCl pH 7.5, 500 mM sodium chloride, 0.05% Tween 20) at room temperature for one hour. The blots were washed twice using TBS-TT (TBS-T containing 0.2% Triton) and once using TBS (10 mM Tris-HCl pH 7.5, 150 mM sodium chloride). Incubation with primary antibody was done overnight at 4°C in 1:10000 dilution of anti-AAG mouse monoclonal (Sigma, Inc., St. Louise, MO, USA) in blocking buffer. The membranes were washed twice with TBS-TT, once with TBS, incubated for two hours with anti-mouse IgG HRP-linked antibody (GE Healthcare, Buckinghamshire, UK) at a 1:5000 dilution in TBS-T, and washed three times with TBS-TT. The blots were developed using diaminobenzidine (DAB) substrate (40 mg/mL DAB, 80 mg/mL nickel chloride, and 30% H_2_O_2 _in 0.1 M Tris-HCl pH 7.5). The stained membranes were then scanned and quantified using ImageJ software (National Institute of Health, Bathesda, MD, USA) by measuring the relative intensity from each band.

### Statistical analysis

Comparison of clinical characteristics, plasma biomarker levels, and Western blot relative intensities among the groups were analyzed using parametric or non-parametric analyses based on data distribution. For normal distributed data, differences between groups were analyzed using Student's *t*-test. For non-parametric distributed data and binary variables, Mann Whitney was performed to compare groups. The descriptive statistics for the markers were compared by scatter plots using the GraphPad Prism 5.02 (GraphPad Inc., CA, USA). To determine the optimal cut-off values for AFP, AAG, and DCP in the diagnosis of HCC, Receiver Operating Characteristic (ROC) curves were constructed using all the possible cut-offs for each assay. The area under the ROC curve (AUC) was calculated and compared. Statistical analysis was performed using the SPSS 15.0 (SPSS Inc., IL, USA).

## Results

### Patient characteristics

A total of 220 patients were included in this study, of which 96 were control and 124 were HCC cases. Of the cases, 61 were AFP-low and 63 were AFP-high. Both the AFP-low and AFP-high HCC patients were older than control (*P *= 0.01). All groups had more male than female patients. The characteristics of these patients were shown in Table 1. The majority of control and cases had a viral etiology of their liver diseases, with HBV in 57 patients out of 96 controls and 71 out of 124 HCC cases, of which 29 were AFP-low HCC. HCV was the underlying etiology of liver disease in 32 patients control and 30 HCC cases of which 12 were AFP-low HCC. Other HCC patients had a non-viral etiology (non B, non C) of their liver diseases, with 20 AFP-low and 3 AFP-high HCC patients.

**Table 1:** Clinical characteristics of patients

Variable	Control (*n *= 96)		HCC (*n *= 124)		*P*-value (vs. control)	
	Chronic Hepatitis (*n *= 49)	Cirrhosis (*n *= 47)	AFP ≤ 20 ng/mL (*n *= 61)	AFP > 20 ng/mL (*n *= 63)	AFP-Low HCC	AFP-high HCC
Age	42.4 ± 14.9	53.8 ± 9.2	55.8 ± 14.6	53.5 ± 12.9	0.0008	0.0127
Gender (M:F)	25:24	31:16	41:20	51:12	0.2659	0.0030
Etiology (%)					0.0124	0.2458
HBV	69.4	48.9	47.5	66.7		
HCV	30.6	36.2	19.7	28.6		
Non HBV-non-HCV	0	14.9	32.8	4.8		
AST (IU/mL)	32.5 (12-194)	42 (16-110)	91 (5-714)	138 (9-945)	0.0026	< 0.0001
ALT (IU/mL)	32.5 (6-868)	32 (15-63)	46 (8-363)	65 (6-347)	< 0.0001	< 0.0001

Data presented as mean ± SD or median (range)

HCC, hepatocellular carcinoma; HBV, hepatitis B virus; HCV, hepatitis C virus; AFP, α-fetoprotein; AST, aspartate aminotransferase; ALT, alanine aminotransferase

### Biomarker levels

**Figure 1:**
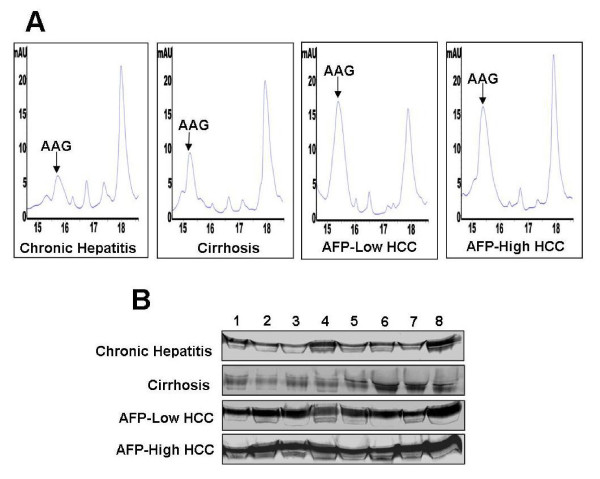
**HPLC chromatogram profile and western blot analysis**. (A) HPLC chromatogram profile of chronic hepatitis, cirrhosis, AFP-low HCC, and AFP-high HCC; (B) Western blot analysis of AAG in chronic hepatitis, cirrhosis, AFP-low HCC, and AFP- high HCC.

HPLC chromatogram analysis showed that peak of AAG at retention time 14.5-16 minute revealed high protein expression in staging of HCC patients with low AFP and high AFP concentration, but this peak was notably smaller in control (Figure 1A). Furthermore, the result from Western blot showed level of AAG in HCC patients with low AFP and high AFP were significantly elevated than those in controls (*P *< 0.001), which was consistent with the result of HPLC (Figure 1B).

**Figure 2:**
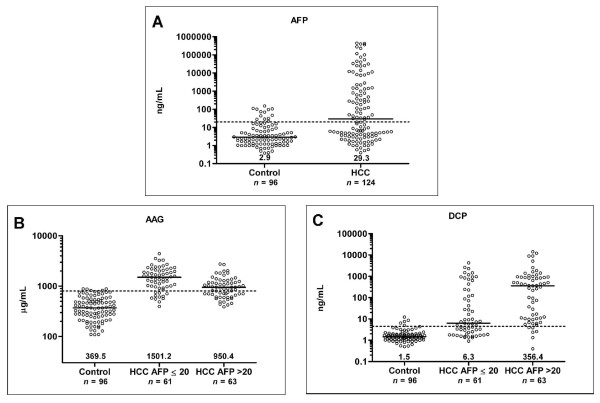
**Scatter plots of plasma AFP, AAG, and DCP**. Scatter plots of plasma (A) AFP, (B) AAG, and (C) DCP levels in control, AFP-low HCC, and AFP-high HCC. The black line indicates the median, for which the value is indicated on the bottom of the scatter plot. The dashed line indicates the cut-off value of each marker, 20 ng/mL for AFP, 800 μg/mL for AAG, and 4.5 ng/mL for DCP. In all markers, *P *< 0.0001 for control vs. either AFP-low HCC or AFP-high HCC.

The AFP, AAG, and DCP values were reported as median because of the skewed data distribution. The AFP level was lower in control than in HCC patients (*P *< 0.0001), with median (range) 2.9 ng/mL (0.4-151.8 ng/mL) and 29.3 ng/mL (0.4-444550 ng/mL), respectively. We observed plasma AFP values above 20 ng/mL in 13 (13.5%) patients of control. Although AFP values among some patients of control were higher than 20 ng/mL, histological investigations showed no HCC. Plasma AAG values were significantly higher in HCC than in control (*P *< 0.0001). The median (range) values for control, AFP-low and AFP-high were 369.5 μg/mL (108.2-883.9 μg/mL), 1501.2 μg/mL (395.6-4419.5 μg/mL) and 950.4 μg/mL (387.2-2748.8 μg/mL), respectively. Significant difference between control and HCC was also observed in DCP values (*P *< 0.0001) with median (range) values were 1.5 ng/mL (0.5-12.3 ng/mL), 6.3 ng/mL (0.9-4273.8 ng/mL) and 356.4 ng/mL (0.4-14245 ng/mL), in control, AFP-low and AFP-high, respectively. Values for AFP, AAG, and DCP were shown in Figure 2.

### Area under the ROC curves

**Figure 3:**
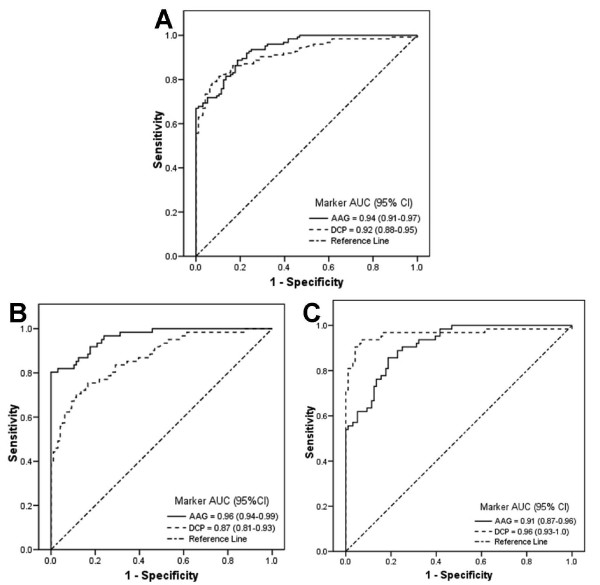
**ROC curves of AAG and DCP in HCC patients**. ROC curves comparing AAG and DCP in (A) all HCC patients; (B) AFP-low HCC patients; and (C) AFP-high HCC patients.

As shown in Figure 3, when all patients with HCC were evaluated, the AUC for total AAG (0.94, 95% CI: 0.91-0.97) was similar to that for DCP (0.92, 95% CI: 0.88-0.95) (*P *= 0.40), but higher than AFP (0.75, 95% CI: 0.69-0.81) (*P *< 0.0001). When AFP-high HCC was compared to control, DCP had higher AUC (0.96, 95% CI: 0.93-1.0) than AAG (0.91, 95% CI: 0.87-0.96; *P *= 0.08). In contrast, when AFP-low HCC was compared to control, AAG had AUC (0.96, 95% CI: 0.94-0.99) better than DCP (0.87, 95% CI: 0.81-0.93; *P *< 0.01) indicating that AAG was more predictive of AFP-low HCC than of AFP-high HCC. Clinical performances of AAG and DCP were shown in Table 2.

**Table 2:** Clinical performance of AAG and DCP at specificity of 95%

Marker	Cut-off	Sensitivity	Accuracy	PPV	NPV
All HCC (*n *= 124)					
AAG	800	71	82	95	72
DCP	4.5	74	83	95	74
AFP-low HCC (*n *= 61)					
AAG	800	82	90	91	89
DCP	4.5	57	80	88	78
AFP-high HCC (*n *= 63)					
AAG	800	62	82	89	79
DCP	4.5	90	93	92	94

PPV, positive predictive value; NPV, negative predictive value; HCC, hepatocellular carcinoma; AAG, α-1-acid glycoprotein; DCP, des-γ-carboxy prothrombin; AFP, α-fetoprotein

### Sensitivity, specificity, accuracy, PPV, and NPV of AAG and DCP

Based on 95% specificity, cut-off for AAG was revised from previous study [14] and increased to 800 μg/mL. Using the cut-off, higher specificity was reached, with sensitivity, accuracy, PPV and NPV were 71%, 82%, 95%, and 72%, respectively. There were 11 (8.9%) AFP-low and 24 (19.4%) AFP-high HCC patients who had AAG values below the cut-off. In addition, there were 5 (5.2%) patients of control who had AAG values above the cut-off.

Cut-off for DCP was taken at 95% specificity on 4.5 ng/mL. In clinical studies of patients with liver disease, instead of using cut-off at 7.5 ng/mL [15], a decision cut-off of 4.5 ng/mL was found to be optimal, giving better clinical performances. The sensitivity, accuracy, PPV, and NPV were 74%, 83%, 95%, and 74%, respectively. Based on the cut-off value, we observed 32 (25.8%) HCC patients had DCP values below the cut-off, while 5 (5.2%) patients of control had values above the cut-off.

In all HCC patients, sensitivity, accuracy, and NPV of DCP were better than those of AAG. However, when ROC analysis was done in AFP-low HCC patients clinical performances of AAG increased over DCP. Sensitivity, accuracy, PPV, and NPV of AAG (82%, 90%, 91%, and 89%, respectively) were better than those of DCP (57%, 80%, 88%, and 78%, respectively). In AFP-high HCC patients, DCP was proven to be better than AAG. The sensitivity, accuracy, PPV, and NPV of DCP were 90%, 93%, 92%, and 94%, respectively, while the corresponding values for AAG were 62%, 82%, 89%, and 79%.

## Discussion

According to the World Health Organization (WHO), the incidence of HCC in Indonesia is projected to steadily increase until 2030 due to increasing frequency of cirrhosis in patients with chronic HBV infection [16]. Surveillance programs have been done based on abdominal ultrasound examination and plasma AFP measurement every six months. However, AFP was found to be a poor complement for ultrasound, because of its low sensitivity and specificity [17], therefore, a search for new biomarkers for HCC is needed.

Several tumor markers have been proposed as complements or substitutes for AFP in HCC diagnosis. Plasma concentration of AAG has been suggested as a potential marker for cirrhosis and HCC [13,14]. A study by Kang et al. found that AAG had similar sensitivity value in differentiating HCC regardless of the tumor size [18]. While in our previous study, the diagnostic efficacy of AAG among the HCC patients with AFP levels below 200 ng/mL was similar to the results from total HCC patients [14]. This previous study clearly demonstrates that the plasma AAG concentration was increased in patients with HCC in comparison to patients with non-HCC. In this study, when the ROC of AAG was compared among HCC patients, the AAG level was significantly higher in HCC patients with low AFP value but lower in HCC patients with high AFP values. These results were supported by another study that mentioned the use of AAG to diagnose HCC patients whose AFP values were below 500 ng/mL [18]. Since AFP level was found to be correlated with later stage and poor prognosis of HCC [19], therefore it allowed us to propose that determining AAG levels could be helpful in early detection of HCC or AFP-low HCC. The high level of AAG in HCC patients with low AFP concentration could be caused by the inflammation of the liver or the overproduction of the acute phase protein by hepatoma cells [12]. However, further study will be required for clarifying the role of AAG in AFP-low HCC.

Several case-control studies showed sensitivities and specificities of different biomarkers in the diagnosis of HCC. A study in Japan of 1377 HCC patients and 355 non-HCC controls with chronic hepatitis or cirrhosis showed that the utility of DCP for the diagnosis of HCC was lower than that of AFP for small tumors, but higher than that of AFP for large tumors [11]. Moreover, DCP level was reported to be closely correlated with tumor progression and prognosis, while HCC with high serum levels of DCP and low levels of AFP were an indication of larger tumor size [20-22]. Similarly, we found that DCP level increased according to the stepwise progression of liver disease, *i.e*., from chronic hepatitis to cirrhosis to HCC. The DCP level was decreased in HCC patients with low AFP value but significantly increased in patients with high AFP value. The sensitivity and specificity were 74% and 95%, respectively, for all HCC patients. These values were similar to the results in China (77% and 86.4%, respectively) [15] but higher than the results in USA (74% and 70%, respectively). Recent study demonstrated that AFP was more sensitive than DCP for the diagnosis of early and very early stage HCC at a new cutoff of 10.9 ng/mL [10]. However, neither AFP alone, DCP alone, nor the combination of AFP and DCP was sufficiently accurate to be used for HCC surveillance [17]. In light of our recent results and others, we propose to use AAG as alternative biomarker to AFP and as a complement for ultrasound in HCC patients with low AFP.

The most important question is whether the cut-off value of DCP in Indonesian HCC patients was similar to studies in other countries. We found the optimal cut-off DCP value to be 4.5 ng/mL based on the ROC curve for distinguishing patients with HCC from those with non-HCC. This DCP cut-off value was lower than the value used in studies from USA (7.5 ng/mL) [15]. This difference might be due to ethnic differences [23] as other studies have also used varying cut-off DCP values for Italian, Japanese and Chinese subjects [19,24]. It is also possible that the etiology of liver disease (HBV and HCV) can alter the DCP level. In this study, HBV was the underlying etiology in most of our patients with chronic hepatitis/cirrhosis and our patients with HCC. Interestingly, we found that the non-viral etiology (non B, non C) among HCC patients was also higher, especially for AFP-low HCC. Taken together, our results suggest that a lower cut-off of DCP value in our study may be related to higher HBV-infected and non B-non C populations among Indonesian subjects. Prospective studies on a large number of patients with diverse ethnic backgrounds and a broad spectrum of underlying etiologies of liver disease are required in order to confirm this assumption.

## Conclusion

Our cross-sectional study showed that AAG was better performance in diagnosing HCC patients with low AFP, while DCP did better in those with high AFP. A DCP value of > 4.5 ng/mL and AAG value of > 800 μg/mL among patients with underlying non-malignant liver disease were associated with a high probability of developing HCC. Prospective studies on a large number of patients with underlying etiologies of liver disease and tumor staging are required to confirm this study. Data from these studies may help to improve the outcome of patients with HCC by enabling the diagnosis to be made at an earlier stage of the disease when curative treatment is still possible.

## Competing interests

The authors declare that they have no competing interests.

## Authors' contributions

IB conceived of, designed, coordinated the study and drafted the manuscript. VK and GAW carried out the AAG and DCP assays and helped with the statistical analysis. RAG, IH, AS, UB, SARL, RM, WA, SS, AS, LAS, and ST coordinated the study and provided the patients sample.
